# Prevention program of emotional and behavioral disorders in children with developmental language delay

**DOI:** 10.1192/j.eurpsy.2022.586

**Published:** 2022-09-01

**Authors:** A. Leonova, T. Raeva

**Affiliations:** Tyumen State Medical University, Psychiatry And Narcology Department, Tyumen, Russian Federation

**Keywords:** prevention, emotional impairments, behavioral impairments, psychoeducation

## Abstract

**Introduction:**

The number of children with developmental language delay is growing. But the isolated use of speech therapy doesn’t always help to improve the long-term prognosis. It was found that developmental language delay is almost never the only violation of a child.

**Objectives:**

To develop the prevention program of emotional and behavioral disorders in children with developmental language delay.

**Methods:**

100 children with developmental language delay (70 boys; *M*age= 26.9 months, *SD* = 5.5) and 50 children with typical language development were studied by clinical follow-up method. The clinical method was supplemented by the Bayley Scale, the Language Development Survey and the Child Behavior Checklist 1½ -5.

**Results:**

A comprehensive children examination revealed developmental language delay risk factors, the psychomotor profile of the children, and the types of emotional and behavioral impairments, which were determined by us as: emotionally labile, emotionally detached and oppositional. The presence of subclinical disorders symptoms in children makes it necessary to carry out preventive measures. The primary prevention consists of pregnancy planning, effective care in pregnancy and childbirth. The secondary prevention aims to early diagnosis of developmental language delay, risk factors assessment of emotional and behavioral disorders and effective methods application of language and psychomotor development correction. Tertiary prevention have to individualized solves the children problems in accordance with revealed types of emotional and behavioral impairments. But the most important part is psychoeducation (special courses for parents and children with developmental language delay).

**Conclusions:**

The prevention program was developed to prevent numerous psychiatric problems in childhood and adolescence.

**Disclosure:**

No significant relationships.

